# The Natural Law of Cure

**Published:** 1898-04

**Authors:** A. P. Betts

**Affiliations:** Woodburn, Ind.


ARTICLE III.

### THE NATURAL LAW OF CURE.

BY A. P. BETTS, M. D., WOODBURN, IND.

---

When the mind is burdened with the cares and perplexities of life, one turns, with a sense of relief and rest, to the contemplation of simple truth. It is with keen pleasure, only sharpened by the former chaotic state of mind, that one searches the hidden depths, unfolding, each moment, new beauties and sublime possibilities. The Christian—who previous to his conversion has been torn and buffeted by conflicting emotions, swayed by falseisms and doctrines, filled with fear and dread—finds rest and peace when he pins his faith on a simple truth, and trusts in the promises of his Maker. Likewise, the intelligent, thoughtful follower of the healing art, surrounded by false isms, lost in the vast wilderness of pills, plasters, and poisons; hounded by deadly baccilli, and treacherous germs; tossed upon the surging billows of unrest and doubt, looks across the wild, tempestuous ocean of theories and dogmas, longing for a glimpse of simple truth on which to cast his anchor of faith. On the horizon he sees rising from this sea of confusion, a haven of rest, built on simple but eternal foundations.

The study of medical history brings to the cheek a blush of shame. Each page is a bad commentary on medical mis-practice; thousands have fallen victims to the vagaries and superstitions of the past; but, as in the foundation of matter, order superseded chaos, so out of the isms, theories, and confusion of antiquity, will rise a system of medical practice founded upon eternal principles of law and truth. "Will rise," did I say? "Has risen" would be better, for in biochemistry I see the haven of rest to the troubled medical mind, a truth which can not be crushed, but will eventually triumph and supersede the theories and vagaries that have so long held arrogant control. To the mind weary of the old regime, longing for progression, and anxious to receive truth, the underlying principles of biochemistry will be found easy of comprehension. As the plant grows—dependent upon the equilibrium of its component parts—so the human system is dependent upon the quantity and equalization of the organic and inorganic constituents. A deficiency of one or more of these will cause a symptom, or cry for the needed food.

It is not my intention to tear down anything good that may be found in any system of medicine; but this is an age of keen investigation, of idol-breaking and truth-finding. He who is afraid to investigate, for fear that some cherished belief will be swept away, is not a true scientist. Age can not sanctify an error. It is with the kindest of feelings toward the science of other schools, and their many brave and noble defenders, that their underlying principles have been questioned, and the law of supplying deficiencies has been accepted and pushed to the front as The Natunal Law of Cure, by which the inorganic cellsalts of the human blood unite with organic matter to form certain material for carrying on life's processes. Biochemistry opens up a new phase of medical science, and the treatment or disease with the inorganic cell-salts is so rational, so in accordance with well-known principles of natural law, that its basic principles need only be presented to the intellect to be understood and adapted. Great truths exist as really before they are discovered as after they are known to the multitude. The vast continent of North America turned its bosom to the summer sun, and changed its garments of green and brown as the seasons passed by, before Columbus set his face " West and West?"

In 1832 we find the following written in *Stapf's Archieves:* " All the essential component parts of the human body are great remedies;" and again, in the same journal, in 1847: " All constituents of the human body act on such organs, principally where they have a function." But it remained for Dr. Schuessler, of Oldensburgh, Germany, to develop these suggestions, and make the idea foreshadowed in them the basis of a new system.

The word biochemistry is formed from *bios* (the Greek for life) and chemistry. Webster defines chemistry as, " that branch of science which treats of the composition of substances, and of the changes which they undergo." Therefore, biochemistry means that branch of science which treats of the composition of living substances; but a more accurate definition is, that branch of science which treats of the composition of the bodies of animals and vegetables, the process by which the various fluids and tissues are formed, the nature and causes of the abnormal condition called disease, and the restoration of health by supplying to the body the deficient cell-salts. The chemical composition of nearly every fluid and tissue of the human body has long been known, but until biochemistry was introduced, no practical use was made of this knowledge in the treatment of the sick.

Biochemistry is science, not experimentation. There is no more of mystery and miracle about it than about all natural laws. An analysis of the blood shows it to contain organic and inorganic matter. The organic constituents are sugar, fats, and abluminous substances. The inorganic constituents are water, and certain minerals, commonly called cell-salts. Of a living, human being, water constitutes over seven-tenths, the cell-salts about one-twentieth, organic matter the remainder. Not until recently were the inorganic cell-salts understood and appreciated. Being little in quantity, they were thought to be little in importance; but now it is known that the cell-salts are the vital portion of the body, the workers, the builders; that the water and organic substances are simply inert matter used by these salts in building the cells of the body. Should a deficiency occur in one or more of these workers, of which there are twelve, some abnormal conditions are known by the general term of disease, and according as they manifest themselves in different ways and in different parts of the body, they have been designated by various names. But these names totally fail to express the real trouble. Every disease which afflicts the human race is due to a lack of one or more of these inorganic workers. Every pain or unpleasant sensation indicates a lack of some inorganic constituent of the blood. Health and strength can only be maintained so long as the system is properly supplied with these cell-salts. Having learned this, it follows, naturally, that the proper method of cure is to supply to the blood that which is lacking. Any disturbance in the molecular motion of these cell-salts in living tissues constituting disease, can be rectified by administering the same cell salt in small quantities, to fill up the gap in the chain of molecules of the affected cell, or tissue salts. This therapeutic procedure is styled, by Schuessler the biochemic method. The blood not being perfectly balanced in all its parts, does not perfectly feed and nourish the nerves, muscles, and other tissues of the body; then a call or dispatch is sent to the throne of understanding, asking that food may be supplied. These words, asking for material with which to carry on the process of life, have, through ignorance, been called disease.

Each mineral salt has a special work to do; each has an affinity for certain organic materials used in building up the human frame. Thus, kali muriaticum, or potassium chloride molecules, work in fibrin. If a deficiency occurs in this salt, a portion of the fibrin not having workers becomes a distributing element, and may be thrown out of the vital circulation, through the nasal passages, lungs, kidneys, or bowels, producing conditions called catarrhs, colds, and coughs.

I have many times tested the efficiency of these cell-salts in disease in the past three years. In fact, my practice is conducted, almost wholly, with these biochemic remedies, and I can say that I have been more than usually successful in my work since I began using them, and I find the practice of medicene more interesting each year as I become more and more acquainted with these remedies. In order to achieve the striking results recorded by the use of these remedies, it is essential to procure these remedies prepared strictly according to the biochemical method; the best preparation of these is in the triturated form, with sugar of milk nine parts, to one part of the salt. It has been my object to give a general idea of this new system of medicine, with the hope of inspiring others to investigate it as I have done. This may be done by procuring some good work upon the subject, such, for instance, as Geo. W. Carry's Biochemic System of Medicine, for sale by F. A. Luyties, of St. Louis.

If the Editor desires I will, at some future time, give the results of my experience with this new system of medicine.—*The Medical Brief.*

